# Functional analysis of alternative splicing of the *FLOWERING LOCUS T* orthologous gene in *Chrysanthemum morifolium*

**DOI:** 10.1038/hortres.2016.58

**Published:** 2016-11-23

**Authors:** Yachao Mao, Jing Sun, Peipei Cao, Rong Zhang, Qike Fu, Sumei Chen, Fadi Chen, Jiafu Jiang

**Affiliations:** 1 College of Horticulture, Nanjing Agricultural University, Nanjing 210095, China

## Abstract

As the junction of floral development pathways, the FLOWERING LOCUS T (FT) protein called ‘florigen’ plays an important role in the process of plant flowering through signal integration. We isolated four transcripts encoding different isoforms of a *FT* orthologous gene *CmFTL1*, from *Chrysanthemum morifolium* cultivar ‘Jimba’. Sequence alignments suggested that the four transcripts are related to the intron 1. Expression analysis showed that four alternative splicing (AS) forms of *CmFTL1* varied depending on the developmental stage of the flower. The functional complement experiment using an *Arabidopsis* mutant *ft-10* revealed that the archetypal and AS forms of *CmFTL1* had the function of complementing late flower phenotype in different levels. In addition, transgenic confirmation at transcript level showed *CmFTL1* and *CmFTL1ast* coexist in the same tissue type at the same developmental stage, indicating a post-transcriptional modification of *CmFTL1* in *Arabidopsis*. Moreover, ectopic expression of different AS forms in chrysanthemum resulted in the development of multiple altered phenotypes, varying degrees of early flowering. We found that an alternative splicing form (*CmFTL1-astE134)* without the exon 2 lacked the ability causing the earlier flower phenotype. The evidence in this study indicates that complex alternative processing of *CmFTL1* transcripts in *C. morifolium* may be associated with flowering regulation and hold some potential for biotechnical engineering to create early-flowering phenotypes in ornamental cultivars.

## Introduction

Alternative splicing (AS) produces multiple mRNA transcripts or isoforms from a single gene through the assembly of exons in different ways using alternative splice sites in the precursor mRNA (pre-mRNA).^1^ AS is known to contribute to gene regulation and proteome diversity, and in plants AS is involved in many important biological processes including stress responses,^2,3^ development^4–6^ and reproductive growth.^7,8^ On the basis of EST/cDNA evidence in *Arabidopsis*, it has been predicted that more than 61% of genes are alternatively spliced under normal conditions, and the number will increase under stress.^9,10^

In *Arabidopsis*, the *FLOWERING LOCUS T* (*FT*) gene has been demonstrated to encode florigen, which integrates environmental and endogenous signals and triggers flowering.^11,12^ The florigen is a small protein (FT) and belongs to a large group of plant proteins with structural similarities to phosphatidylethanol-amine-binding protein. In *Arabidopsis*, FT proteins move to the shoot apical meristem via the phloem, where they interact with a basic leucine zipper (bZIP) transcription factor, FD to form a transcriptional complex. This complex directly activates floral regulator genes such as *APETALA 1* (*AP1*) and *FRUITFULL* (*FUL*) to promote flowering.^13,14^ In general, FT orthologous genes consist of four exons and three introns and have a high level of sequence homology and similar functions in evolutionarily distant plant species.^15–17^ AS of *FT* orthologous genes was previously observed in London plane (*Platanus acerifolia* Willd), maize (*Zea mays*) and sunflower (*Helianthus annuus*). In the *Zea mays, two orthologous FT genes ZCN18 and ZCN26 (Zea mays CETRORADIALIS)*, have developmentally specific patterns with alternatively spliced transcripts.^18^ The alternative splicing of *PaFT* in *Platanus acerifolia* was more complicated in terms of temporal and spatial expression patterning, suggesting it may be associated with multiple regulatory roles in different stages of growth.^19^ In sunflower, a frameshift mutation in the third exon of *HaFT1* affects flowering time through interference with the function of another paralog.^20^

Most chrysanthemum (*Chrysanthemum morifolium*) cultivars are typical short-day plants where the flowering time is subject to the short-day conditions. Stable year-round flower production is costly and difficult. In recent years, deeper understanding of the mechanisms of floral transition at the molecular level and further developments in chrysanthemum are a point of interest to researchers. Three *FT-like* (*FTL*) genes have been isolated from a wild diploid chrysanthemum, *Chrysanthemum seticuspe*. CsFTL3 acts as a floral stimulator under SD conditions while the functions of other two *CsFTLs* are still not clear.^21^ Currently, AS activity in *FT* orthologous genes have never been reported in chrysanthemum. In the present work, *CmFTL1* and four isoforms resulting from alternative splicing were isolated from *C. morifolium* cultivar ‘Jimba’. Detailed sequencing, splicing sites and types of *CmFTL1* alternatively spliced forms were studied. Ectopic expression of *CmFTL1* and four AS transcripts were examined for complementation of the *Arabidopsis ft-10* mutants. The phenotypes of chrysanthemum that overexpressed *CmFTL1* and *CmFTL1ast*s showed varying degrees of early flowering. The results provided strong evidences of highly functional conservation of *FT* orthologs in both *Arabidopsis* and chrysanthemum.

## Materials and methods

### Plant material and growth conditions

*Chrysanthemum morifolium* cultivars ‘Jimba’ were used for the experiments. Plants were grown in a greenhouse and maintained at 20 °C with an 8 h photoperiod (SD conditions) and the light intensity of 1200 μmol m^−2^ s^−1^. To analyse the expression patterns of *CmFTL1* and *CmFTL1asts* under natural conditions, leaf samples were initially collected at an early developmental stage and at 3-day intervals until visual colour stage, then samples were collected at 5-day intervals until flowers opened. All leaf samples were collected at 16:00 hours^21–23^ on each time point.

For genetic transformation experiments, transformed *Arabidopsis* plants were grown in a greenhouse with a 16 h photoperiod (LD condition) and a 25 °C/18 °C day/night temperature. Transgenic chrysanthemum plants were grown under the condition described above.

### *CmFTL1* and *CmFTL1asts* cloning

Total RNA was extracted from the third fully expanded leaf (counting from the apex) of ‘Jimba’ plants using the RNAiso reagent (TaKaRa, Dalian, China) and treated with RNase-free DNase (TaKaRa) according to the manufacturer’s instructions. The cDNA synthesized from a 1 μg aliquot of total RNA by using the TaKaRa RNA PCR Kit (AMV) v2.1 (TaKaRa) was used as a template to isolate both the *CmFTL1* and *CmFTL1ast* sequences by performing a PCR primed by the oligomer pair 5′-TAATGCCGAGGGAAAGGGAT-3′/5′-GTCATGATTGATTCAGTTAAAGTGT-3′. Full-length versions of the cDNAs were derived by applying both 5′- and 3′-RACE PCR using a GeneRacer kit (Invitrogen, Carlsbad, CA, USA). The amplicons (~500 bp in length) were inserted into the pMD19-T vector (TaKaRa) for sequencing. Genomic DNA was extracted from juvenile leaves using a modified CTAB method.^24^ The first intron (intron#1) sequence of CmFTL1 was amplified by a PCR based on the primer pair 5′-GCGTAATAGGGGATGTTCTTGATAG-3′/5′-ACTAGGACTTGGAGCATCAGGAT-3′. The resulting amplicon was introduced into pMD19-T for sequencing.

### *CmFTL1* and splicing sites analysis

The sequence of the *CmFTL1* gene was submitted to NCBI and analysed with BLAST (http://blast.ncbi.nlm.nih.gov/Blast.cgi). The genomic DNA sequence of *CmFTL1* exon 1–intron 1–exon 2 was submitted to the Alternative Splice Site Predictor (http://wangcomputing.com/assp/) to enable a prediction of the AS sites present. The full-length mRNA of *CmFLT1* was submitted to NCBI (http://blast.ncbi.nlm.nih.gov/Blast.cgi) for BLASTing, and the sequences of homologous genes in other species with high identities were identified. The multiple sequences alignment was performed using the software BioXM2.6 (Nanjing Agricultural University, Nanjing, China) and DNAMAN (Lynnon LLC., San Ramon, CA, USA).

### Expression analysis by quantitative real-time PCR and semi-quantitative PCR

The abundance of *CmFTL1* transcripts was determined by semi-quantitative PCR and quantitative real-time PCR (qRT-PCR). Total RNA was extracted using the RNAiso reagent (TaKaRa) followed by RNase-free DNase treatment (TaKaRa). A 1 μg aliquot of total RNA was reverse-transcribed with SMART MMLV Reverse Transcriptase (Clontech, Palo Alto, CA, USA) according to the manufacturer’s instructions. The resulting cDNA was diluted fivefold of its original concentration, and a 5 μL aliquot was used as template in a 15 μL qPCR SYBR Premix Ex Taq (TaKaRa). All reactions were performed in triplicates. The reaction was initially denatured (95 °C/2 min), then cycled 40 times through 95 °C/15 s, 55 °C/15 s, 72 °C/20 s. The chrysanthemum *EF1a* sequence (AB679278) was chosen as the reference. The ‘calibrator sample’ was designated as the most highly expressed time point for each gene of interest and therefore represented the highest relative transcription level of 1.0. The data showed the mean and s.e. of at least three biological replicates. The qRT-PCR template was a 1 μL aliquot of cDNA (1000 ng μL^−1^); the reaction conditions were given in Supplementary Table 2.

### *Arabidopsis* and chrysanthemum transformation

Both the *A. thaliana FT* loss-of-function mutant *ft-10* and ‘Jimba’ were transformed with various *CmFTL1* constructs.

The *A. thaliana FT* promoter (6977 nt) from the pENTR4-*AtFT* vector was sub-cloned into pHGW (Invitrogen) to obtain pHGW-*proAtFT* ^25^ via LR recombination. The *CmFTL1* open reading frame was amplified from the cDNA described above with the primer pair (Supplementary Table 1), the product was then inserted into pHGW-*proAtFT* to form pHGW-*proAtFT-CmFTL1*. This construct was used to test for complementation of the *A. thaliana ft-10* mutant. To construct the *CmFTL1* overexpression vector, the open reading frame sequence of *CmFTL1* and *CmFTL1asts* was amplified using a pair of primers with *Sal*I and *Not*I restriction enzymes sites (Supplementary Table 1), and the resulting PCR product was digested with corresponding restriction enzymes and inserted into pENTR1A, then sub-cloned into the GATEWAY vector pMDC43^26^ via LR recombination. *Arabidopsis* plants were transformed using the floral dip method,^27^ while transgenic lines of *C. morifolium* ‘Jimba’ were achieved using a modified *Agrobacterium*-mediated transformation protocol.^28^

## Result

### Molecular cloning and sequence analysis of *CmFTL1* and *CmFTL1ast*s from ‘Jimba’

**Figure 1:**
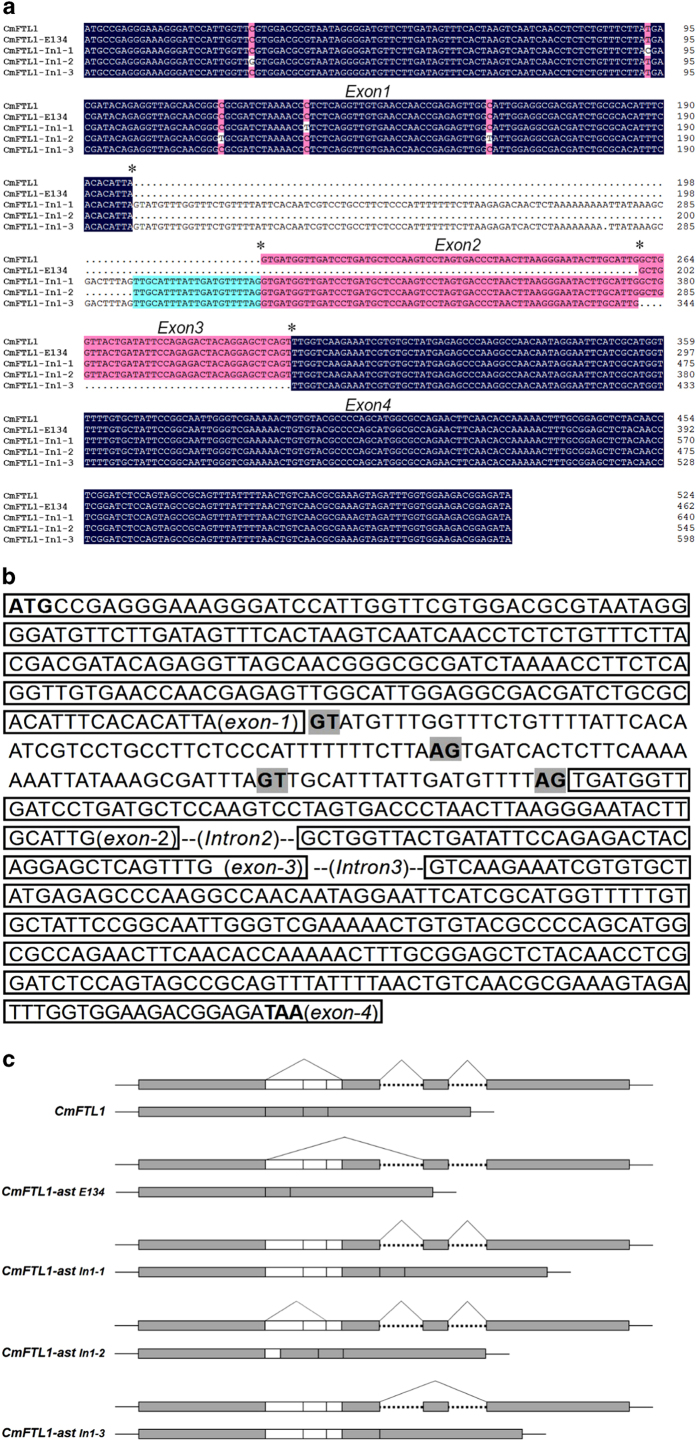
The nucleotide sequences of *CmFTL1* and its AS products. (**a**) Sequence alignment of *CmFTL1* cDNA and its AS products; asterisks indicate separation of the four exons. (**b**) The genomic sequence of a segment of *CmFTL1*. Exons are shown boxed. The letters highlighted in grey indicate the AS sites. The bold letters indicate the initiation (ATG) and the termination (TAA) codons. (**c**) Processing of *CmFTL1*. Horizontal lines indicate the 5′ and 3′ UTRs. The coding regions are shown as grey-stippled boxes and the introns as white boxes; dotted lines indicate unknown introns. The vertical lines in the white boxes indicate the AS sites.

We used RACE strategies to clone the *CmFTL* genes of *C. morifolium* cultivar ‘Jimba’. The products of *CmFTL1* were electrophoresed through 2% agarose gels and cloned into the sequencing vector. Different sizes of *CmFTL1* cDNA products were obtained. Analysis of the sequences revealed that the *CmFTL1* had at least five different alternatively spliced transcripts. Compared with the normal full-length *CmFTL1* transcript, two of the alternatively spliced products (*CmFTL1astIn1-1*, *CmFTL1astIn1-2*) contained the different lengths of the intron and two transcripts (*CmFTL1astE134* and *CmFTL1astIn1-3*) lacked exons (Figure 1a).

To confirm the *CmFTL1* alternative splicing sites, the intron1 was cloned from gDNA and sequenced (Supplementary Figure 1). The prediction of AS sites by ASSP showed the sites at boundary of intron1 are not only the normal GT-AG constitutive splicing sites, but also had the alternatively spliced potential. The intron1of *CmFTL1* had two latent alternative acceptor sites (Figure 1b). According to reports,^29^ there are five distinct types of alternative splicing events: exon skip type (ES type), intron retention type (RI type), alternative donor type (AD type), alternative acceptor type (AA type) and mutually exclusive exons (MEE type). Besides the archetypal spliced form (*CmFTL1*), four other *CmFTL1* spliced forms were detected (Figure 1c). Among the four spliced forms, *CmFTL1-astE134* and *CmFTL1-astIn1-3* were resulted from ES type events, while *CmFTL1-astIn1-1* and *CmFTL1-astIn1-3* were derived from RI type events. *CmFTL1-astIn1-2* had only an AD type event. Comparison of the various *CmFTL1* splice forms showed that alternative splicing events tended to occur more frequently in the first intron than in the second or third intron.

### The polypeptide sequence of *CmFTL1* and *CmFTL1ast* transcripts

**Figure 2:**
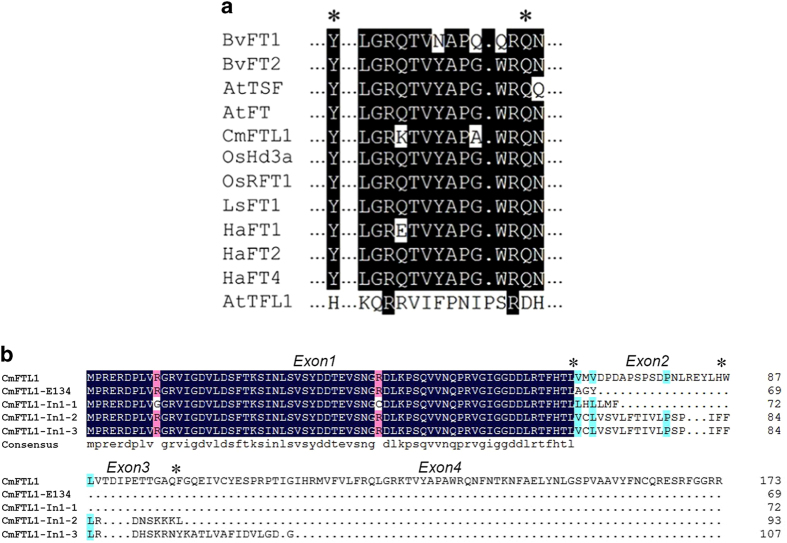
Analysis of the amino-acid alignments of *CmFTL1* and *CmFTL1asts*. Multiple alignment on GENEBANK: *AtFT* (AB027504.1), *AtTFL1* (NM_120465.2), *AtTSF* (GQ884981.1), *BvFT1* (HM448910.1), *BvFT2* (HM448912.1), *HaFT1* (GQ884981.1), *HaFT2* (GQ884987.1), *HaFT4* (GU985573.1), *LsFT* (BAK14368.1), *OsHd3a* (JX472280), *OsRFT1* (AB426873.1). (**a**) Partial amino-acid sequence alignment of the phosphatidylethanol-amine-binding protein (PEBP) family members. Asterisks indicate the residues Tyr85 (Y)/Gln140 (Q) and His88 (H)/Asp144 (D) contributing to FT and TFL1 functioning, respectively.^30, 31^ The conserved segment region B in the fourth exon, corresponding to the external loop of the PEBP family proteins, is boxed. (**b**) Alignment of amino-acid sequence of CmFTL1 and its alternative splicing transcripts, the asterisks separate the exons into Exon1, Exon2, Exon3 and Exon4.

The archetypal *CmFTL1* transcript encodes a 174 residue protein which shares 72% sequence identity with the *A. thaliana* FT protein, 90% with lettuce LsFT and 89% with rice RFT1 (Table 1). The residues Tyr85 and Gln140, both of which are critical for the functionality of AtFT,^30,31^ were present in CmFTL1, at positions 84 and 139, respectively (Figure 2a). Those AS transcripts in which the number of nucleotides inserted/deleted was not a multiple of three are expected to encode a frame shift product, while those truncated by the creation of a premature termination codon are expected to have lost residues in exon 4 essential for FT-like activity^30,31^ (Figure 2b).

Alternative splicing plays an important role in increasing the diversity of proteomes in both animals and plants. AS forms encode variant proteins, leading to completely different or multiple biological functions. However, the number of nucleotides inserted/deleted in *CmFTL1* alternative splicing forms were not in multiples of 3, resulting in frame-shifts that truncated the CmFTL1asts protein and losing the amino acids that are critical for FT or TFL1-like protein function and the regions that are essential for FT-like activity in exon 4^30,31^ (Figure 2b). In addition, premature termination codons (PTCs) may be the targets for nonsense-mediated mRNA decay (NMD),^32,33^ however many PTC-containing transcripts also exist that remain functional.^34,35^ Thus, further research of the protein encoded by *CmFTL1asts* is required to confirm whether the PTC-containing transcripts lead to functional truncated proteins or to NMD.

### Phenotypes of transgenic *Arabidopsis* expressing ectopic *CmFTL1* and *CmFTL1asts*

In order to gain the functional divergence of *CmFTL1* and its alternative splicing isoforms, we attempted to rescue the loss-of-function *A. thaliana* mutant (*ft-10*) by *CmFTL1* and *CmFTL1asts*, respectively. The vectors carrying *AtFTpro*: *CmFTL1* and *35S: CmFTL1asts* were separately introduced into *A. thaliana* by *Agrobacterium*-mediated transformation (Supplementary Figure 2).

**Figure 3:**
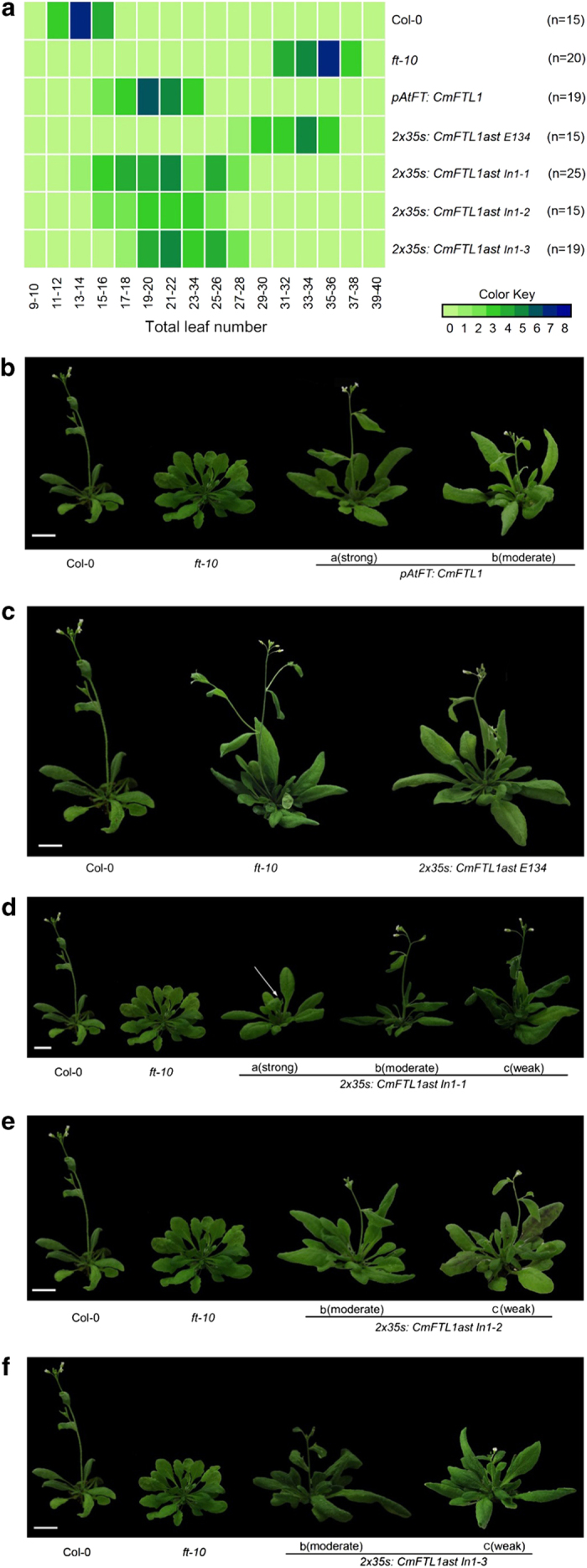
The phenotypic effect of expressing *CmFTL1-*derived transgenes in *A. thaliana*. (**a**) Rosette leaf number at the flowering stage of transgenic and non-transgenic *A. thaliana*. Plants grown under long day conditions. (**b**–**f**) The phenotype of transgenic plants either harbouring or not harbouring *AtFT,* and constitutively expressing (**b**) *CmFTL1,* (**c**) *CmFTL1astE134,* (**d**) *CmFTL1astIn1-1*, (**e**) *CmFTL1astIn1-2* or (**f**) *CmFTL1astIn1-3*. (**a**) Strong complementation (13–18 rosette leaves) at flowering, (**b**) moderate complementation (19–24), (**c**) weak complementation (>25). Bar, 1 cm.

The effect on phenotype of the various transgenes was assessed by comparing the number of rosette leaves formed at the flowering stage between the transgenic lines constructed in both a wild-type and a *ft-10* mutant background. Under the growing conditions imposed, wild-type plants produced 15 rosette leaves at flowering and the *ft-10* mutants 36. On the basis of the number of rosette leaves produced at the flowering stage, we classified the recovery phenotypes of transgenic lines as strong (<18), moderate (19–24) and weak (>25). Among 19 *proAtFT*::*CmFTL1 ft-10* transformants analysed, 5 produced strong phenotype, while the other 14 produced moderate phenotypes (Figure 3a). Thus the *CmFTL1* transgene appeared able to at least partially overcome the delay in flowering induced by the loss of *AtFT* (Figure 3b). Three of the four *CmFTL1ast* sequences (the exception was *CmFTL1astE134*) were able to partially rescue the *ft-10* mutant. Among 25 transformants with the *35S:CmFTL1-ast In1-1* in *ft-10* mutant, 8 lines displayed strong phenotype, 11 lines with moderate phenotype and the remaining 6 lines displayed weak recovery (Figure 3c). Among a total of 15 transgenic lines with *35S:CmFTL1-ast In1-2* transgenes, 4 lines showed strong changes in flowering time (13–18 rosette leaves), 9 lines showed moderate changes and the rest 2 lines showed weak recovery change in flowering time (Figure 3d). Among 15 of the *35S:CmFTL1-ast In1-3* transgenic lines, only 1 line showed strong phenotype, 12 lines showed the moderate phenotype and the rest 8 lines showed weak phenotype (Figure 3e).

To verify that the the various *CmFTL1-*derived transgenes were successfully transcribed in *A. thaliana*, the putative transgenic lines were tested by qRT-PCR. In the lines harbouring an AS product, fragments corresponding to various sizes were detected (Supplementary Figure 3). Sequencing of the amplicons showed that the *CmFTL1astIn1-1*, *1-2* and *1-3* lines each produced AS as well as post-transcriptionally modified forms, suggesting that the intron1-containing AS products were processed post-transcriptionally to recreate the archetypal *CmFTL1* transcript able to promote flowering.

### Expression patterns of *CmFTL1* and *CmFTL1asts* in chrysanthemum

**Figure 4:**
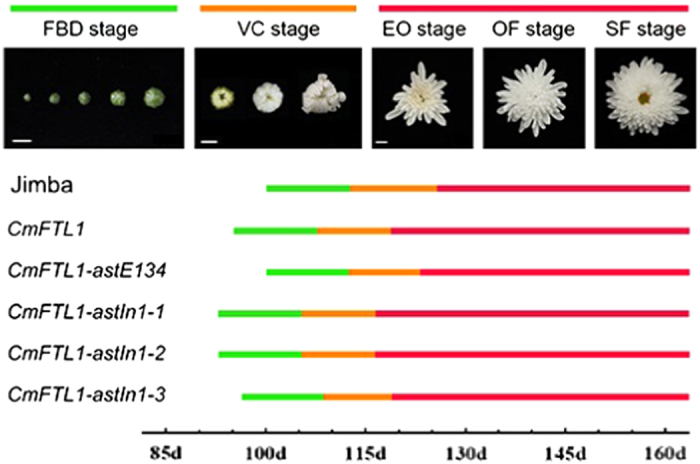
The floral transition in chrysanthemum wild type and *CmFTL1* transgenic lines. EO, early opening stage; FBD, flower bud development stage; OF, open flower stage; SF stage, senescent flower stage; VC, visible colour stage. Scale Bars, 1 cm.

**Figure 5:**
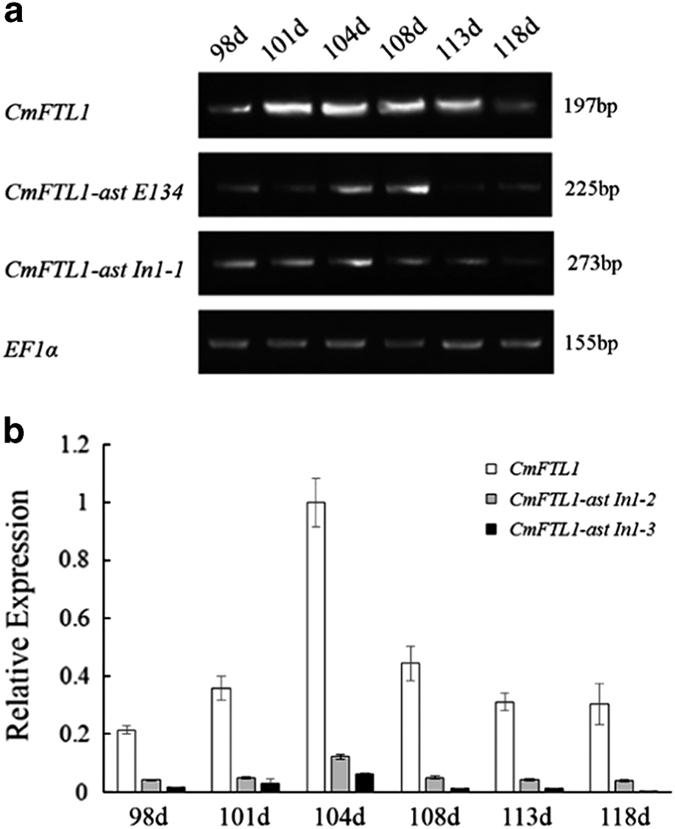
The transcription of *CmFTL1* and its AS products in the leaf of wild-type chrysanthemum plants grown under natural conditions. (**a**) The transcription of *CmFTL1astsE134* and *CmFTL1astIn1-1* was assayed using qRT-PCR. (**b**) The transcription of *CmFTL1astIn1-2* and *1-3* was assayed using qPCR. Time points were days after transplanting. 98d: prior to the FBD stage; 101–104d: the FBD stage; 104–108d: the VC stage; 108–118d: the EO stage. Transcript abundances were normalized against that of *CmEF1a*.

**Figure 6:**
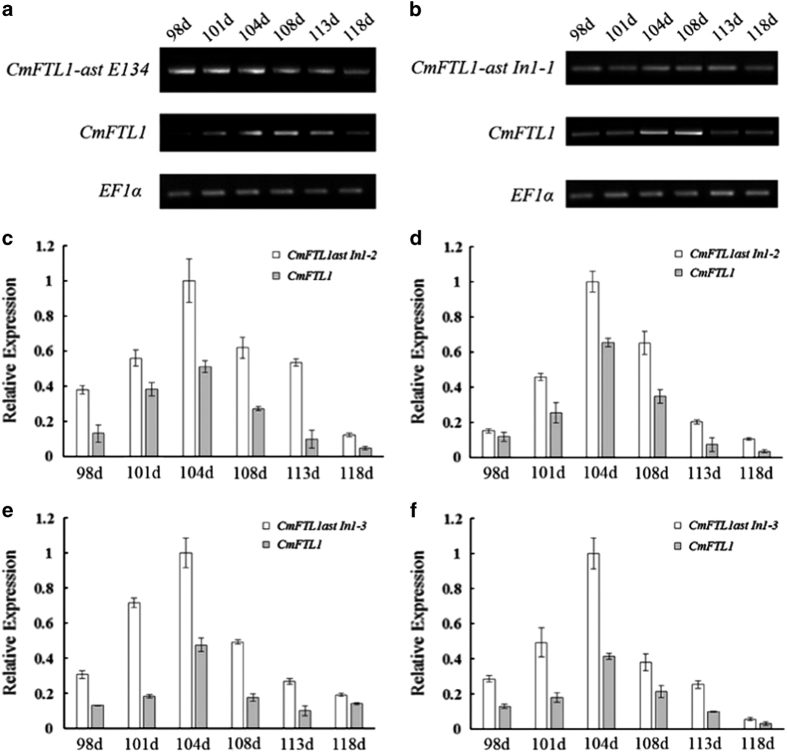
The transcripts of *CmFTL1* in its AS products transgenic chrysanthemum plants. (**a**) *CmFTL1astsE134* line 49; (**b**) *CmFTL1astIn1-1* line 63; (**c**) *CmFTL1astIn1-2* line 4; (**d**) *CmFTL1astIn1-2* line 10; (**e**) *CmFTL1astIn1-3* line 4; (**f**) *CmFTL1astIn1-3* line 25. Time points were days after transplanting. 98d: prior to the FBD stage; 101–104d: the FBD stage; 104–108d: the VC stage; 108–118d: the EO stage. The transcription of *CmFTL1astsE134* and *CmFTL1astIn1-1* was assayed using qRT-PCR, and that of *CmFTL1astIn1-2* and *1-3* by qPCR.

To investigate the potential function of *CmFTL1* and *CmFTL1asts* in chrysanthemum, transgenic chrysanthemum plants with *CmFTL1* and *CmFTL1asts* expressed under the control of the *Cauliflower mosaic virus* (CaMV) *35S* promoter were generated, named by OX-*CmFTL1* or OX-*CmFTL1asts* (Supplementary Figures 4–6). The phenotypes of transgenic lines were observed under natural conditions. The flower development of chrysanthemum involves six stages: before flower bud development stage, flower bud development stage (FBD), visible colour stage (VC) stage, earlier opening stage, opened flower stage and senescing flower stage.^22^ The FBD and the VC stage of OX-*CmFTL1astIn1-1* and OX-*CmFTL1astIn1-2* lines were both advanced by 5–8days, while OX-*CmFTL1astIn1-3* lines were advanced only by 2–4 days. No significant alteration to flowering time was noted in the transgenic plants constitutively expressing *CmFTL1astE134* and wild-type plants (Figure 4). The qRT-PCR and qPCR analyses showed that in wild-type plants, the abundance of *CmFTL1* transcript peaked at the early flower bud development stage (Figure 5). Although the temporal pattern of the AS forms was similar to the wild type, their transcript abundance was much lower. However, the AS transcripts were higher than the *CmFTL1* in the AS transgenic chrysanthemum plants (Figure 6). The results suggested that the *CmFTL1* AS transcripts underwent post-transcriptional processing, similar to the *CmFTL1ast A. thaliana* transgenic lines, resulting in an increased abundance of the archetypal transcript.

## Discussion

AS represents a significant strategy for regulating gene expression, through the generation of a diversity of proteins. The investigation of AS in *A. thaliana* over the past decade has revealed that almost half of the genes are affected.^36^ Transcriptomic analysis has suggested that >61% of *A. thaliana* genes may contain the intron-generated AS products. The proportion will likely rise as a greater range of developmental stages and growing conditions will be explored in the future.^10,37^ The product of *FT* and its various homologues is the key to regulate the timing of the transition from vegetative to reproductive growth. Here we demonstrated that the chrysanthemum *FT* homologue generated various forms of AS, as do the *FT* homologues in maize, sunflower and the London plane.^18,19,38^ The individual AS products of the maize genes *ZCN18* and *ZCN26* have not been defined, while the sunflower gene *HaFT1* produces a transcript containing a part of its third intron, introducing premature stop codons. The London plane *FT* gene displays a rather complicated AS profile.

The AS products of *CmFTL1* involved ES-, RI- and AD-type events. Four of the splicing points occurred in intron1 even though this sequence is just 118 nt in length. The equivalent intron in *AtFT* (817 nt) is much longer than this, so intron length is clearly not a decisive factor in determining AS frequency. Some experimental evidences gathered from rice and *A. thaliana* suggest that preferential combinations of AS events exist,^25,39^ but the data obtained here from chrysanthemum are insufficient to imply any such preference. The multiple alternative splicing forms of *PaFT* (*Platanus acerifolia*) show that the ES type is always associated with the AD type.^19^ Our data show that two of *CmFTL1asts* have the similar preference of alternative splicing types. The intron splice boundaries generally follow the rule of ‘GT…AG’.^40^ The alternative splicing sites tends to weaker than the constitutive splicing sites so that the sites in introns or exons that have alternative splicing events are not typical.^41^ The predicted position of the AS sites in intron 1 of *CmFTL1* suggested that intron splice boundary sequences need not be the expected GT and AG, but can also have features of AS sites, suggesting that the AS sites in *CmFTL1* are not randomly placed, but more likely reflect the outcome of directed evolutionary selection. The results would imply that the AS messages are functional. For example, in the case of the gene *FLC*, which encodes a repressor of flowering involved in the vernalization pathway, AS sites are shared between the two highly disparate species canola (oilseed rape, *Brassica rapa*) and trifoliate orange (*Poncirus trifoliata*).^7,42^ It is clear that the *FT* sequence are largely conserved during the evolution of the angiosperms, and that it tends not to experience frequent AS events. So far there were only three plant species that *FT* orthologs involved AS, suggesting that the selection of AS sites in *CmFTL1* is an atypical *FT* orthologs.

In chrysanthemum, as in rice,^23^ FTL1 shows weak florigenic activity under short day conditions. The potential regulatory control of *CmFTL1* over floral transition was explored by constitutively expressing the sequences corresponding to both the archetypal *CmFTL1* transcript and its four AS forms in both *A. thaliana* and chrysanthemum. The ability of CmFTL1 to rescue the phenotype of the *ft-10* mutation established that the chrysanthemum homologue has an equivalent functionality to the native AtFT. The partial rescue of the *ft-10* mutant phenotype achieved by constitutively expressing three of the four AS sequences suggested that post-transcriptional processing was able to convert them into the archetypal *CmFTL1* transcript. The behaviour of the transgenic chrysanthemum lines constitutively expressing *CmFTL1* and its various AS products was similar to the transgenic *A. thaliana* plants. A possible scenario is that the *CmFTL1* AS transcript encodes a truncated form of CmFTL1, which shows attenuated florigenic activity; alternatively, its product may induce the interaction of the CmFTL1 with CmFDL1, which induces flowering in both chrysanthemum and the *A. thaliana ft-10* mutant. Because many AS transcripts also exist and remain functional,^34,35^ we hypothesize that the *CmFTL1asts* may have opportunities to occurred post-transcriptional modification when lacking of *CmFTL1* at floral transition and formed the archetypal to complement the function of *CmFTL1*. And both *A. thaliana ft-10* mutant and chrysanthemum that ectopically expressed *CmFTL1astsE134* failed to show any florigentic activity, suggesting that the loss of exon2 in *CmFLT1*may influenced its function and then affected the regulation of flowering timing.

In non-transgenic chrysanthemum plants, the abundance of *CmFTL1* transcript was substantially greater than that of the other four AS transcripts. This relationship was altered in the transgenic plants in which *CmFTL1* or one of its AS products was constitutively expressed, suggesting that the AS transcripts were processed post-transcriptionally, as was the case in the transgenic *A. thaliana* plants. Thus, our data demonstrate that both *CmFTL1* and its AS forms are involved in the regulation of floral transition. The florigenic activity of the *CsFTL1* product is weaker than that of *CsFTL3* when these genes are expressed in the chrysanthemum variety ‘Jimba’ under long days.^21,23^ Although residual CsFTL3 and increased CsFTL1 activity are likely to have a major impact on the flowering process, the phyB-mediated protein CsAFT is also known to be important in determining the flowering response to photoperiod in chrysanthemum.^23^ This may explain why CmFTL1 shows such a low level of florigen in ‘Jimba’.

In summary, this study showed that *CmFTL1* is somewhat atypical of *FT* homologues, since its transcript can be modified prior to its translation. The participation in the flowering process of both *CmFTL1* and its AS forms was demonstrated by studying the phenotypic consequences of expressing these genes constitutively in both *A. thaliana* and chrysanthemum. The representation of the various transcriptional forms of the gene was dependent on the developmental stage of the plant, but some post-transcriptional modification of the AS products narrowed the gap of transcription levels. The results demonstrate that manipulating *CmFTL1* may have potential benefit in shortening the life cycle of the chrysanthemum plant.

## Figures and Tables

**Table 1:** Peptide identity among FTL1 homologues

*(Identities) %*	*AtFT*	*BvFT1*	*BvFT2*	*CmFTL1*	*Hd3a*	*LsFT*	*RFT1*
AtFT		69.83	75.43	72	69.83	74.86	67.98
BvFT1			81.56	70.39	67.4	71.67	65.19
BvFT2				77.14	77.09	80.68	76.97
CmFTL1					75.42	90.29	73.03
Hd3a						78.77	88.83
LsFT							75.84
