# Supplementary material for: Functional analysis of alternative splicing of the FLOWERING LOCUS T orthologous gene in Chrysanthemum morifolium
Source: Hortic Res. 2016 Nov 23;3:16058–. doi: 10.1038/hortres.2016.58 (PMC5120556; doi:10.1038/hortres.2016.58)
Supplement: Supplementary Information [file hortres201658-s1.docx]

**Supplement Information**

Supplementary Table 1. Primers for the PCR amplification in this study.

| Primer name | Sequence（5'-3'） | Remarks |
| --- | --- | --- |
| E134-F | TTGCGGCCGCTAATGCCGAGGGAAAGGGAT | Forward primer of full-length *CmFTL1ast E134* |
| E134-R | TGATGTGCGTGCTTTCAAAATATGC | Reverse primer of full-length *CmFTL1ast E134* |
| In11-F | TTGCGGCCGCTAATGCCGAGGGAAAGGGAT | Forward primer of full-length *CmFTL1ast In1-1* |
| In11-R | TATGCTTGTAACATCCTCTTCATGC | Reverse primer of full-length *CmFTL1ast In1-1* |
| In12/3-F | ATGCCGAGGGAAAGGGATCCATTG | Forward primer of full-length *CmFTL1ast In1-2*/*3* |
| In12/3-R | TATGCTTGTAACGTCCTCTTCATGC | Reverse primer of full-length *CmFTL1ast In1-2*/*3* |
| meiqFTL1-F | CCGCTCGAGATGCCGAGGGAAAGGGAT | Forward primer of full-length *CmFTL1* contains restriction sites of *Xho*Ⅰ |
| meiqFTL1-R | AAAACTGCAGTTATCTCCGTCTTCCACCAA | Reverse primer of full-length *CmFTL1* contains restriction sites of *Pst*Ⅰ |
| meiqAS-F | CGAGCTCATGCCGAGGGAAAGGGAT | Forward primer of full-length *CmFTL1asts* contains restriction sites of *Sal*Ⅰ |
| meiqE134-R | ATAAGAATGCGGCCGCGATGTGCGTGCTTTCAA | Reverse primer of full-length *CmFTL1ast E134* contains restriction sites of *Not*Ⅰ |
| meiqIn11-R | ATAAGAATGCGGCCGCTATGCTTGTAACATCCTCTTCATGC | Reverse primer of full-length *CmFTL1ast In1-1* contains restriction sites of *Not*Ⅰ |
| meiqIn12-R | ATAAGAATGCGGCCGCAACGTCCTCTTCATGC | Reverse primer of full-length *CmFTL1ast In1-2* contains restriction sites of *Not*Ⅰ |
| meiqIn13-R | ATAAGAATGCGGCCGCTATGCTTGTAACGTCCTCTT | Reverse primer of full-length *CmFTL1ast In1-3* contains restriction sites of *Not*Ⅰ |
| RT- E134-F | ATGACGATACAGAGGTTAGCAACGGGC | Forward primer for RT-PCR |
| RT-E134-R | GCACACGATTTCTTGACCAAACTGAG | Reverse primer for RT-PCR |
| RT-In11-F | CAATCAACCTCTCTGTTTCTTACGA | Forward primer for RT-PCR |
| RT-In11-R | CACGATTTCTTGACCAAACTGAGC | Reverse primer for RT-PCR |
| qIn12-F | GACGATACAGAGGTTAGCAACGGGT | Forward primer for qRT-PCR |
| qIn12-R | AATAATGTGTGAAATGTGCGCAGAT | Reverse primer for qRT-PCR |
| qIn13-F | GCATTGGTCAAGAAATCGTGTG | Forward primer for qRT-PCR |
| qIn13-R | AGTTTTTGGTGTTGAAGTTCTGGC | Reverse primer for qRT-PCR |
| *CmEF1α*-F | TTTTGGTATCTGGTCCTGGAG | Forward primer of reference genes in *C. morifolium* |
| *CmEF1α*-R | CCATTCAAGCGACAGACTCA | Reverse primer of reference genes in *C. morifolium* |

Supplementary Table 2. Semi-quantitative real-time PCR and conditions used in the study.

| **Gene** | **PCR conditions** |
| --- | --- |
| *CmFTL1* | A |
| *CmFTL1-ast E134* | A |
| *CmFTL1-ast In1-1* | A |
| *CmFTL1-ast In1-2* | A |
| *CmFTL1-ast In1-2* | A |
| *CmEF1α* | B |

A) PCR was performed with an initial denaturing step at 94 ̊C for 3 min, followed by 28 cycles at 94 ̊C for 30 sec, 55 ̊C for 30 sec, 72 ̊C 30 for sec; and 72 ̊C for 10 min.

B) PCR was performed with an initial denaturing step at 94 ̊C for 3 min, followed by 22 cycles at 94 ̊C for 30 sec, 55 ̊C for 30 sec, 72 ̊C 30 for sec; and 72 ̊C for 10 min.

**
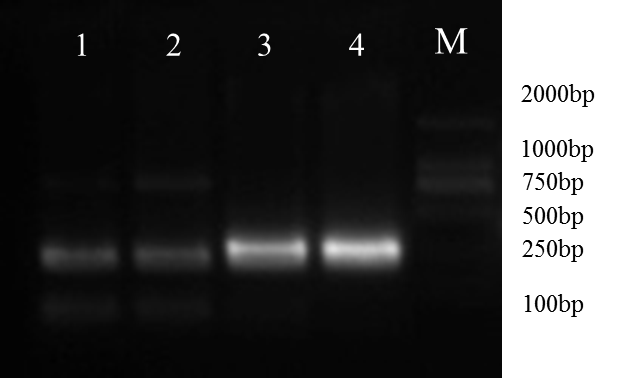
**

**Supplementary Fig 1.** Agarose gel electrophoresis of *CmFTL1* intron1. 1&2, 3&4 products of two different primers, the length is 100~250bp; M. DL2000


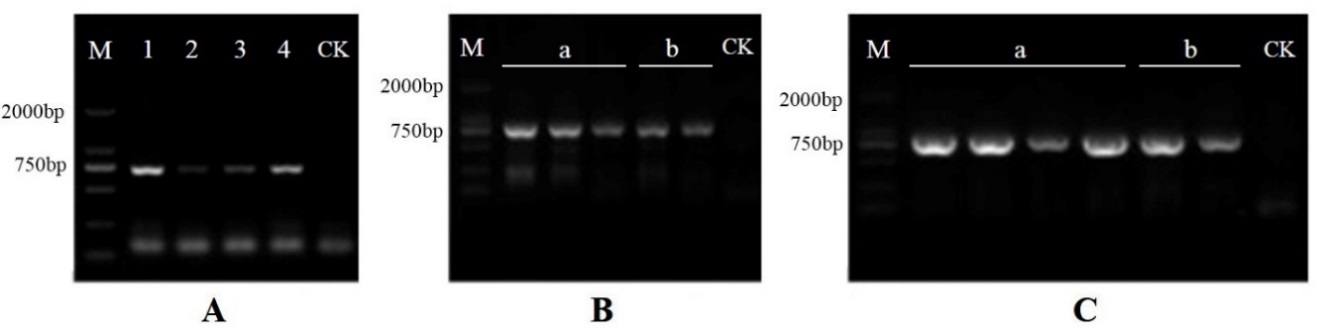


**Supplementary Fig 2.** PCR detection of the resistance lines. A: *FTpro::CmFTL1*; B: a. *CmFTL1astE134*, b. *CmFTL1astIn1-2*; C: a. *CmFTL1astIn1-1*, b. *CmFTL1astIn1-3*; CK: Non-transgenic *ft-10*; PCR production of Hyg resistance gene is 750bp; M: DL2 000 DNA Marker.


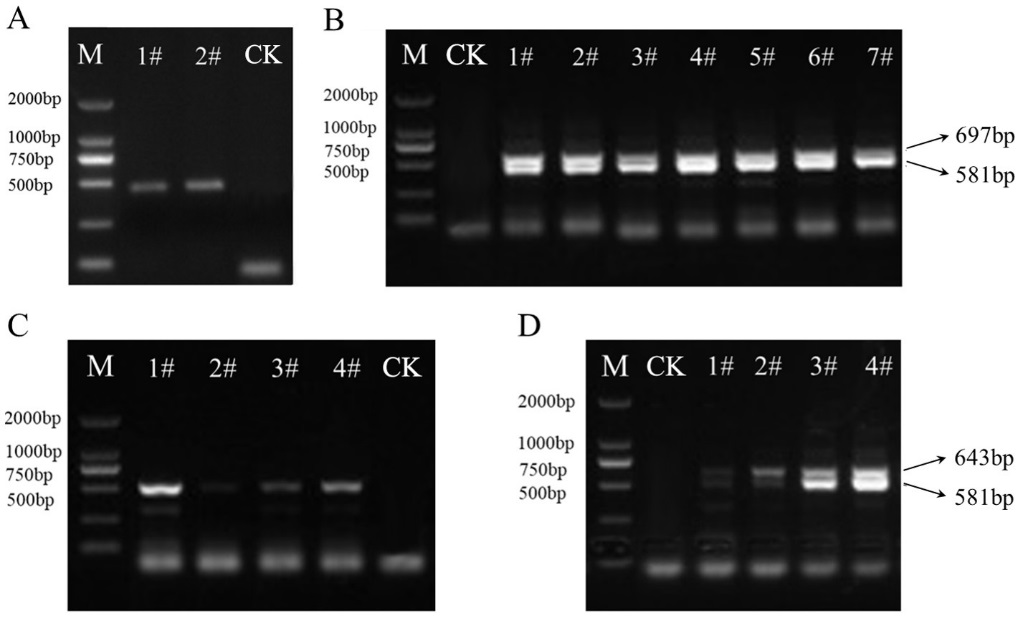


**Supplementary Fig 3.** Validation of the transcription of *CmFTL1ast* transgenes in *A. thaliana.*

1. *CmFTL1astE134,* (B) *CmFTL1astIn1-1,* (C) *CmFTL1astIn1-2*, (D) *CmFTL1astIn1-3*. M: DNA size ladder (Marker2000).

**
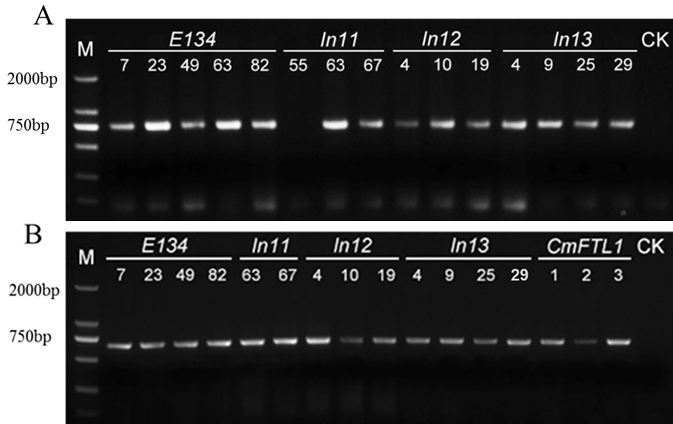
**

**Supplementary Fig 4.** PCR confirmation of Hyg resistance lines at DNA level

A: PCR detection of hygromycin gene from ‘Jimba’OX-*CmFTL1asts* transgenic plant, production is 750bp; B: PCR detection by using vector-gene primers from ‘Jimba’OX-*CmFTL1asts* transgenic plant, production is 700bp; CK is non-transgenic ‘Jimba’;

DL2 000DNA Marker; different numbers represent transgenic lines separately.


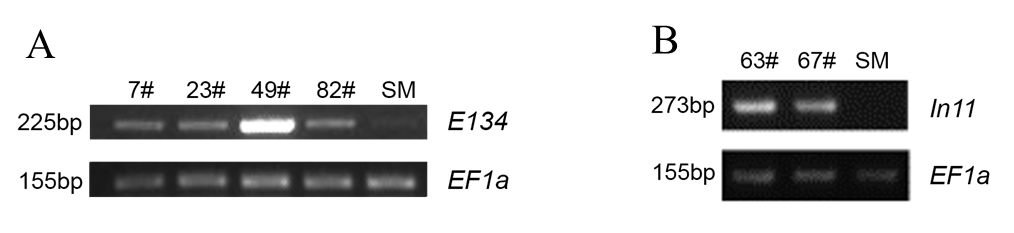


**Supplementary Fig 5.** RT-PCR detection of *CmFTL1astE134* and *CmFTL1astIn1-1* in transgenic chrysanthemum. Fig. A represents for over-expressed *CmFTL1astE134* transgenic plants, Fig. B represents for over-expressed *CmFTL1astIn1-1* transgenic plants. CK was non-transgenic control plants, numbers representing for transgenic lines.


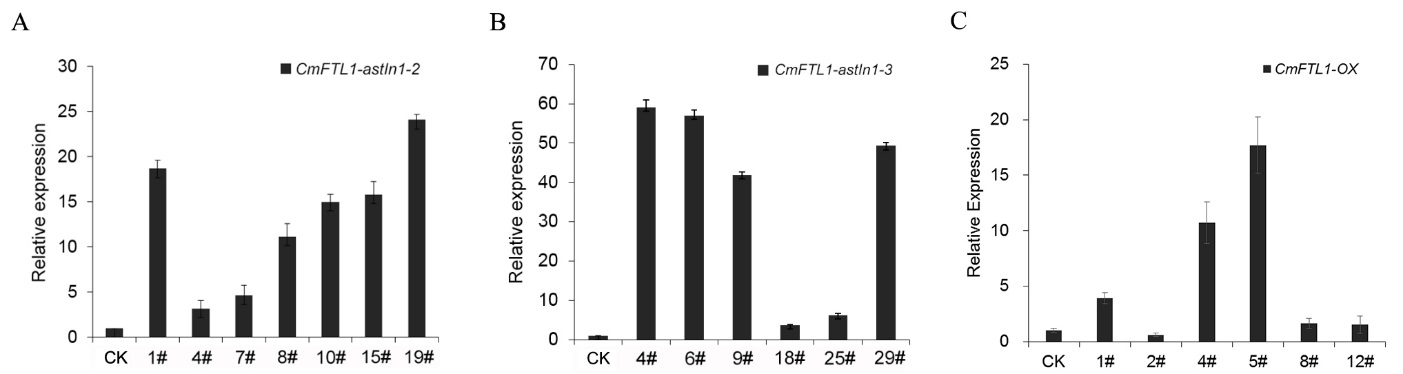


**Supplementary Fig 6.**  qRT-PCR detection of *CmFTL1astIn1-2* and *CmFTL1astIn1-3* in transgenic lines. Fig. A represents for over-expressed *CmFTL1astIn1-2* transgenic plant. Fig. B represents for over-expressed *CmFTL1astIn1-3* transgenic plant. Fig. C represents for over-expressed *CmFTL1* transgenic plant.CK was non-transgenic control plants.
